# Pediatric biorepository participation during the COVID-19 pandemic: predictors of enrollment and biospecimen donation

**DOI:** 10.1186/s12887-022-03185-6

**Published:** 2022-03-12

**Authors:** Anne M. Neilan, Anisha Tyagi, Yao Tong, Eva J. Farkas, Madeleine D. Burns, Allison Fialkowski, Grace Park, Margot Hardcastle, Elizabeth Gootkind, Ingrid V. Bassett, Fatma M. Shebl, Lael M. Yonker

**Affiliations:** 1 Division of General Academic Pediatrics, Department of Pediatrics, Boston, MA USA; 2 Division of Infectious Disease, Department of Medicine, Boston, MA USA; 3 Massachusetts General Hospital, Medical Practice Evaluation Center, 100 Cambridge Street, Floor 16, Boston, MA USA; 4 Harvard Medical School, Boston, MA USA; 5 Massachusetts General Hospital, Department of Pediatrics, Pulmonary Division, Boston, MA USA; 6 Massachusetts General Hospital, Mucosal Immunology and Biology Research Center, Boston, MA USA

**Keywords:** COVID-19, Pediatrics, Biorepository, Consent, Children, Adolescent

## Abstract

**Background:**

Patient-level predictors of enrollment in pediatric biorepositories are poorly described. Especially in pandemic settings, understanding who is likely to enroll in a biorepository is critical to interpreting analyses conducted on biospecimens. We describe predictors of pediatric COVID-19 biorepository enrollment and biospecimen donation to identify gaps in COVID-19 research on pediatric biospecimens.

**Methods:**

We compared data from enrollees and non-enrollees aged 0–25 years with suspected or confirmed COVID-19 infection who were approached for enrollment in the Massachusetts General Hospital pediatric COVID-19 biorepository between April 12, 2020, and May 28, 2020, from community or academic outpatient or inpatient settings. Demographic and clinical data at presentation to care were from automatic and manual chart extractions. Predictors of enrollment and biospecimen donation were assessed with Poisson regression models.

**Results:**

Among 457 individuals approached, 214 (47%) enrolled in the biorepository. A COVID-19 epidemiologic risk factor was recorded for 53%, and 15% lived in a US Centers for Disease Control and Prevention-defined COVID-19 hotspot. Individuals living in a COVID-19 hotspot (relative risk (RR) 2.4 [95% confidence interval (CI): 1.8–3.2]), with symptoms at presentation (RR 1.8 [95% CI: 1.2–2.7]), or admitted to hospital (RR 1.8 [95% CI: 1.2–2.8]) were more likely to enroll. Seventy-nine percent of enrollees donated any biospecimen, including 97 nasopharyngeal swabs, 119 oropharyngeal swabs, and 105 blood, 16 urine, and 16 stool specimens, respectively. Age, sex, race, ethnicity, and neighborhood-level socioeconomic status based on zip code did not predict enrollment or biospecimen donation.

**Conclusions:**

While fewer than half of individuals approached consented to participate in the pediatric biorepository, enrollment appeared to be representative of children affected by the pandemic. Living in a COVID-19 hotspot, symptoms at presentation to care and hospital admission predicted biorepository enrollment. Once enrolled, most individuals donated a biospecimen.

**Supplementary Information:**

The online version contains supplementary material available at 10.1186/s12887-022-03185-6.

## Introduction

Throughout the COVID-19 pandemic, there has been a paucity of research among school-age children, even though many mitigation strategies disproportionately affect youth (e.g., closing schools but opening non-essential businesses in some settings) [1, 2]. Despite vaccination rolling out among those ≥12 years of age in the fall of 2021, cases among children and adolescents continue to rise, especially in unvaccinated children [3]. The impact of COVID-19 in pediatric populations likely depends on host susceptibility, symptoms, viral load, inoculum, social contact patterns, and behaviors. Studies assessing age-dependent susceptibility to and transmission of COVID-19 have multiple limitations [4]. Research with stored pediatric biospecimens has unique potential to inform key questions about immune responses to COVID-19 infection and inform further research about drivers of the pandemic. In contrast to adult biorepositories, pediatric biorepositories face distinct challenges to consent and thus representative inclusion in research; participants are minors and are considered a vulnerable population, and third-party approval is needed (i.e., from legal guardians) [5]. Enrolling participants that are representative of the pediatric population is essential for the validity, reliability, and interpretation of studies using collected samples. Most research efforts have focused on the effects of the SARS-CoV-2 virus on adult and elderly populations experiencing higher rates of severe illness and mortality [6, 7]. However, pediatric populations are susceptible to severe complications of COVID-19 including multisystem inflammatory syndrome and death, as well as morbidity from long-term COVID-19 symptoms [8].

At the start of the COVID-19 pandemic, a pediatric biorepository was established at Massachusetts General Hospital (MGH) in Boston, Massachusetts [9]. This biorepository collected, processed, and stored donated biospecimens including nasopharyngeal and oropharyngeal swabs, blood, sputum, stool, and urine. We hypothesized that among those approached for enrollment, due to normative development, school-age children (5–12 years) might be less likely to enroll in the biorepository or subsequently donate biospecimens compared to young children and infants (< 5 years) and adolescent and young adults (> 12 years). We also hypothesized that overall willingness to enroll may be higher than in pediatric biorepository studies conducted outside of a pandemic setting. We describe predictors of patient enrollment to a pediatric biorepository to identify strengths and gaps in COVID-19 research on pediatric biospecimens.

## Methods

### Study population

The study population included children and young adults (0–25 years) in community or academic outpatient or inpatient settings with suspected or confirmed COVID-19 infection. Enrollment procedures have been previously described and were conducted verbally (English/Spanish or with the assistance of an interpreter) in the setting of the pandemic [9], based on outpatient clinic appointment schedules or emergency department and hospital admission lists. Individuals were approached for enrollment to the pediatric biorepository at MGH regardless of later COVID-19 test results. To offset the surge in medical demand from older adults during the early phases of the COVID-19 pandemic, pediatric clinics evaluated young adults through age 25. An institutional community research program was also in place at community enrollment sites.

### Clinical and laboratory data

Data were derived from manual chart extractions and automatic electronic data downloaded at presentation to care for both enrollees and non-enrollees who were approached during the first wave of the COVID-19 pandemic in Boston between April 12, 2020, to May 28, 2020. Presentation to care was considered a visit via telephone, video, or in-person. Data included recruitment site, demographics, epidemiological data, and clinical characteristics (vital signs and medical history). Epidemiologic risk was defined by chart review and included international or domestic travel, occupational exposure (non-healthcare), having a household contact, being undomiciled, having received healthcare in a facility with known COVID-19 cases, or other risk factors.

### Study outcomes

The primary outcome variable was whether the participant enrolled in the biorepository. Secondary outcomes included biospecimens donated by enrollees including nasopharyngeal and oropharyngeal swabs, blood, stool, and urine. Urine and stool samples were only collected from inpatients.

### Predictors

Demographic and clinical predictors were evaluated as well as three additional variables: US Centers for Disease Control and Prevention (CDC) hotspot county definition (a minimum of 5 criteria based on prevalence and changes in incidence of COVID-19) [10], COVID-19 county risk level defined by cases per 100,000 people per day by the Harvard Global Health Institute (low < 1; mild: 1–9; moderate: 10–24; and high: ≥25) [11], and Neighborhood Deprivation Index as outlined by Messer et al, which used variables indicative of neighborhood-level socio-economic status based on zip code [12]. The estimated Neighborhood Deprivation Index is a standardized score that has a mean of zero and standard deviation of one. Higher values indicate a more affluent “less deprived” neighborhood (i.e., a neighborhood that has Neighborhood Deprivation Index score of 0.8 indicates a less deprived neighborhood than one with a score of 0.2; likewise a neighborhood with a score of − 0.2 indicates a less deprived neighborhood than one with a score of − 0.8.). Details of these variables are outlined in the Supplemental Methods.

### Statistical analysis

Data are presented as percentages for categorical variables and as median ± inter-quartile range (IQR) for continuous variables. We performed bivariate associations to determine the association between predictors and outcome variables. We compared categorical variables using Chi-squared or Fisher exact tests and continuous variables using Kruskal Wallis tests.

We used multivariable Poisson regression models to assess relationships between outcomes and potential predictors, reporting relative risks (RR), 95% confidence intervals (CI), and *p*-values. Model selection procedure is described in the Supplemental Methods. We conducted principal component analysis to estimate Neighborhood Deprivation Index (Supplemental Methods). All statistical analyses were conducted using SAS version 9.4.

### Institutional review board approval

Research was approved by the Mass General Brigham Human Research Committee (Protocol 2020P003588 and IRB#2020P000955) and the Partners/Massachusetts General Hospital Institutional Biosafety Committee (IBC#2020B000061).

## Results

### Characteristics of the study population

Among 457 individuals approached for enrollment, 46% were female, 28% self-reported as White/non-Hispanic, 6% Black/non-Hispanic, 9% Other, 12% not reported; 44% reported Hispanic ethnicity, and 68% reported English as a primary language (Table 1). The age strata of those approached for enrollment included: 35% 0–5-years-old, 27% 6–12-years-old, 22% 13–18-years-old, and 17% 19–25-years-old. The median (IQR) of Neighborhood Deprivation Index among those approached for enrollment was − 0.6 (− 0.8, 0.7). Most participants were either in home daycare or cared for by a nanny (21%) or in grade school (39%). An epidemiologic risk factor for COVID-19 was noted on chart review among 53% at the time of enrollment; of those, 48% recorded a household contact, with 30% and 8% of household contacts being with parents and siblings, respectively. Only 15% lived in a COVID-19 hotspot as defined by CDC, and none lived in a high COVID-19-risk level county per the Harvard Global Health Institute definition; 87% lived in low COVID-19-risk level counties.

**Table 1:** Baseline characteristics among youth ages 0 to 25 years with suspected COVID-19 approached to participate in a pediatric biorepository in the greater Boston area

Characteristic	Total ^a^ (*n* = 457)	
Sex assigned at birth		
Female	211	(46)
Race/Ethnicity ^b^		
White/non-Hispanic	130	(28)
Black/non-Hispanic	29	(6)
Hispanic	202	(44)
Other	40	(9)
Not reported	56	(12)
Primary language		
English	311	(68)
Spanish	108	(24)
Other	19	(4)
Not reported	19	(4)
Age, median (IQR)	9.1	(3.0–15.7)
Age group		
0–5 years	158	(35)
6–12	124	(27)
13–18	99	(22)
19–25	76	(17)
Neighborhood Deprivation Index, median (IQR)	−0.6	(− 0.8–0.7)
Daycare / school		
Nanny / home daycare	98	(21)
Group daycare	34	(7)
Preschool / kindergarten	25	(5)
Grade school	179	(39)
College	22	(5)
Not reported	99	(22)
Known epidemiologic risk ^c,d^		
Yes	243	(53)
Household contact	219	(48)
Parent	137	(30)
Sibling	35	(8)
Occupational exposure	20	(4)
Undomiciled	2	(1)
Received care in a facility with COVID cases	2	(< 1)
Biogen contact	2	(< 1)
Other	63	(14)
Missing	25	(5)
COVID-19 hotspot	70	(15)
COVID-19 risk level		
Low	397	(87)
Mild	29	(6)
Moderate	31	(7)
High	0	(0)
Site of presentation		
Outpatient clinic	381	(83)
Emergency Department	27	(6)
Other	49	(11)
Hospital admission		
Admitted	44	(10)
Unknown	12	(3)
Symptomatic at presentation to care		
Yes	371	(81)
Missing	6	(1)
Temperature > 98.6 °F		
Yes	162	(35)
Missing	137	(30)
Oxygen saturation ≤ 94%		
Yes	8	(2)
Missing ^e^	156	(34)
Any past medical history	241	(53)
Prescribed any medication		
Yes	195	(43)
Missing	5	(1)
Prescribed immunosuppressant medication		
Yes	3	(1)
Missing	15	(3)
Prescribed asthma medication at presentation to care		
Yes	66	(14)
Missing	1	(< 1)
Prescribed inhaled steroids	20	(4)
Cardiac or Metabolic medical history		
Yes	92	(20)
Missing	13	(3)
If Cardiac or Metabolic = Yes: ^f^		
Dyslipidemia	13	(14)
Obesity	73	(79)
Other	27	(29)
Pulmonary medical history		
Yes	118	(26)
Missing	14	(3)
If Pulmonary = Yes: ^f^		
Asthma	72	(61)
History of pneumonia	13	(11)
Obstructive sleep apnea	16	(14)
Reactive airway disease	7	(6)
Kidney or Liver medical history		
Yes	11	(2)
Missing	14	(3)
History of cancer		
Yes	3	(1)
Missing	15	(3)
Rheumatic or autoimmune medical history		
Yes	5	(1)
Missing	19	(4)
Neurologic or neurodevelopmental condition		
Yes	107	(23)
Missing	20	(4)
History of neurologic events		
Yes	36	(8)
Missing	14	(3)
History of neuro-developmental conditions		
Yes	90	(20)
Missing	20	(4)
Appropriate development for age		
Yes	385	(84)
Missing	20	(4)
History of transplant		
Yes	1	(< 1)
Missing	14	(3)
Preterm delivery < 37 weeks		
Yes	34	(7)
Missing	166	(36)
COVID-19 test conducted	403	(88)
COVID-19 positive among those with COVID-19 test conducted	117	(29)
COVID-19 positive among symptomatic at presentation to care	99	(30)
COVID-19 positive among asymptomatic at presentation to care	18	(26)

Most individuals who were approached for enrollment presented to an outpatient clinic (83%); only 10% of those approached were admitted to the hospital. At the time of presentation, 81% were symptomatic and 35% reported fever. Only 2% presented with oxygen saturation ≤ 94%. Most (53%) reported having any past medical history and 43% reported being prescribed any medication. Among those with a cardiac or metabolic medical history (20%), 79% had obesity. Among those with pulmonary medical history (26%), 61% reported asthma. Eighty-four percent had development appropriate for age recorded, and 20% had a documented neurodevelopmental condition. Of those with a history of neurologic events (8%), most had seizure (47%) and/or headache (19%). There were few individuals prescribed immunosuppressing medications (≤1%) or inhaled steroids (4%). Among those approached for enrollment, COVID-19 testing was conducted for 88%; of these individuals who received a COVID-19 test, 29% had a positive COVID-19 test. Among those who were symptomatic or asymptomatic at presentation to care, 30 and 26% were COVID-19 positive, respectively.

### Characteristics of enrollees versus non-enrollees

Enrollees and non-enrollees had similar distributions of sex assigned at birth, race/ethnicity, primary language spoken, categorical age, oxygen saturation at presentation to care, and most measures of past medical history and COVID-19 testing and positivity (Table 2). Of those approached, 47% enrolled. Enrollees were more likely than non-enrollees to be older (median 10.3 years vs. 8.7 years, *p* = 0.03). Those in preschool/kindergarten (52%) were more likely to enroll in the biorepository than other daycare or school types; those belonging to group daycare (26%) were least likely to enroll (*p* < 0.001). Participants living in a COVID-19 hotspot county were also more likely to enroll (99% vs. 37%, *p* < 0.0001). Participants living in counties at higher COVID-19 risk levels were also more likely to enroll than those at lower risk levels (moderate: 100%; mild: 93%; low: 39%, *p* < 0.0001). Individuals presenting to the Emergency Department or who were transferred from an outside hospital or elsewhere (63% and 75%) were more likely to enroll than individuals presenting to outpatient clinics (42%, *p* < 0.0001). Those admitted to the hospital were more likely to enroll than those who were not admitted (75% vs. 43%, *p* < 0.001). Those who had any symptoms or temperature > 98.6 degrees Fahrenheit at presentation to care were more likely to enroll than those without symptoms (51% vs. 31%, *p* < 0.001; 57% vs. 41%, *p* = 0.004). In terms of past medical history and medication, participants who had asthma (54% vs. 35% *p* = 0.04), took asthma medications (61% vs. 45%, *p* = 0.02) or inhaled steroids (70% vs. 46% *p* = 0.03) were more likely to enroll. Sex assigned at birth, Neighborhood Deprivation Index, and primary language spoken at home were not associated with enrollment.

**Table 2:** Comparison of characteristics of enrollees and non-enrollees in a pediatric biorepository

Characteristic	Enrolled ^a^ (*n* = 214)		Did not enroll ^a^ (*n* = 243)		*P*-value
Sex assigned at birth					0.97
Female	99	(47)	112	(53)	
Male	115	(47)	131	(53)	
Race/Ethnicity ^b^					0.28
White/non-Hispanic	54	(42)	76	(58)	
Black/non-Hispanic	11	(38)	18	(62)	
Hispanic	105	(52)	97	(48)	
Other	20	(50)	20	(50)	
Not reported	24	(43)	32	(57)	
Language					0.76
English	143	(46)	168	(54)	
Spanish	55	(51)	53	(49)	
Other	8	(42)	11	(58)	
Not reported	8	(42)	11	(58)	
Age, median (IQR)	10.3	(3.7–16.6)	8.7	(2.7–14.4)	0.03
Age group					0.06
0–5 years	67	(42)	91	(58)	
6–12	57	(46)	67	(54)	
13–18	44	(44)	55	(56)	
19–25	46	(61)	30	(39)	
Neighborhood Deprivation Index, median (IQR)	−0.6	(−0.8, 0.6)	−0.6	(− 0.8, 0.8)	0.46
Neighborhood Deprivation Index ^c^					0.67
More deprived	110	(48)	120	(52)	
Less deprived	104	(46)	123	(54)	
Daycare / school					< 0.001
Nanny / home daycare	43	(44)	55	(56)	
Group daycare	9	(26)	25	(74)	
Preschool / kindergarten	13	(52)	12	(48)	
Grade school	77	(43)	102	(57)	
College	8	(36)	14	(64)	
Not reported	64	(65)	35	(35)	
Known epidemiologic risk					0.30
Yes	115	(47)	128	(53)	
No	99	(52)	90	(48)	
Household contact					0.74
Yes	103	(47)	116	(53)	
No	99	(46)	117	(54)	
Unknown/Missing	12	(55)	10	(45)	
Household contact: Parent					0.44
Yes	60	(44)	77	(56)	
No	154	(48)	165	(52)	
Household contact: Sibling					0.72
Yes	18	(51)	17	(49)	
No	196	(47)	225	(53)	
COVID-19 hotspot					< 0.0001
Yes	69	(99)	1	(1)	
No	145	(37)	242	(63)	
COVID-19 risk level					< 0.0001
Low (green)	156	(39)	241	(61)	
Mild (yellow)	27	(93)	2	(7)	
Moderate (orange)	31	(100)	0	(0)	
High (red)	0	(0)	0	(0)	
Site of presentation					< 0.001
Outpatient clinic	160	(42)	221	(58)	
Emergency Department	17	(63)	10	(37)	
Hospital transfer or other	37	(76)	12	(24)	
Hospital admission					< 0.001
Admitted	33	(75)	11	(25)	
Not admitted	174	(43)	227	(57)	
Unknown	7	(58)	5	(42)	
Symptomatic at presentation to care					< 0.001
Yes	189	(51)	182	(49)	
No	25	(31)	55	(69)	
Missing	0	(0)	6	(100)	
Temperature > 98.6F					0.004
Yes	93	(57)	69	(43)	
No	64	(41)	94	(59)	
Missing	57	(42)	80	(58)	
Oxygen saturation ≤ 94%					0.07
Yes	7	(88)	1	(13)	
No	135	(46)	158	(54)	
Missing ^d^	72	(46)	84	(54)	
Prescribed any medication					0.05
Yes	104	(53)	91	(47)	
No	108	(42)	149	(58)	
Missing	2	(40)	3	(60)	
Prescribed asthma medication at presentation to care					0.02
Yes	40	(61)	26	(39)	
No	174	(45)	216	(55)	
Missing	0	(0)	1	(100)	
Prescribed inhaled steroids					0.03
Yes	14	(70)	6	(30)	
No	200	(46)	237	(54)	
COVID-19 test conducted					0.62
Yes	187	(46)	216	(54)	
No	27	(50)	27	(50)	
COVID-19 positive					0.71
Yes	56	(48)	61	(52)	
No	131	(46)	155	(54)	
Past medical history					0.98
Yes	113	(47)	128	(53)	
No	101	(47)	115	(53)	
Cardiac or Metabolic					0.56
Yes	43	(47)	49	(53)	
No	163	(46)	189	(54)	
Missing	8	(62)	5	(38)	
If Cardiac or Metabolic = Yes:					
Obesity					0.03
Yes	30	(41)	43	(59)	
No	13	(68)	6	(32)	
Pulmonary					0.17
Yes	55	(47)	63	(53)	
No	149	(46)	176	(54)	
Missing	10	(71)	4	(29)	
If Pulmonary = Yes:					
Asthma					0.04
Yes	39	(54)	33	(46)	
No	16	(35)	30	(65)	
History of pneumonia					0.25
Yes	8	(62)	5	(38)	
No	47	(45)	58	(55)	
Obstructive sleep apnea					0.81
Yes	7	(44)	9	(56)	
No	48	(47)	54	(53)	
Kidney or Liver					0.18
Yes	3	(27)	8	(73)	
No	202	(47)	230	(53)	
Missing	9	(64)	5	(36)	
Neurologic or neurodevelopmental condition					0.23
Yes	51	(48)	56	(52)	
No	150	(45)	180	(55)	
Missing	13	(65)	7	(35)	
History of neurologic events					0.07
Yes	22	(61)	14	(39)	
No	183	(45)	224	(55)	
Missing	9	(64)	5	(36)	
History of neuro-developmental conditions					0.25
Yes	42	(47)	48	(53)	
No	159	(46)	188	(54)	
Missing	13	(65)	7	(35)	
Appropriate development for age					0.28
Yes	185	(48)	200	(52)	
No	19	(37)	33	(63)	
Missing	10	(50)	10	(50)	
Preterm delivery < 37 weeks					0.37
Yes	15	(44)	19	(56)	
No	114	(44)	143	(56)	
Missing	85	(51)	81	(49)	

^a^ n (%), unless otherwise indicated

^b^ Race and ethnicity were electronic medical record self-reported data from registration information

^c^ The median value of Neighborhood Deprivation Index among the whole sample was used to categorize individuals to two categories

^d^ Individuals with missing vitals and other information may have presented for testing only

^e^ At the early stages of this analysis, universal masking had not yet been implemented in health care settings

^f^ More than one category may apply to an individual

### Multivariable regression

**Fig. 1:**
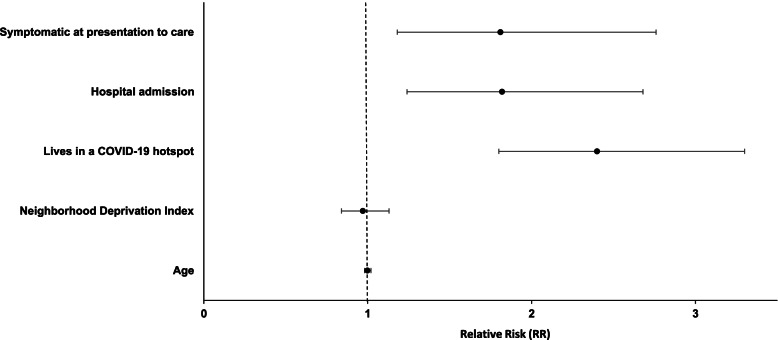
Relative risks of predictors of enrollment. The forest plot shows the relative risk (horizontal axis) and 95% confidence interval (horizontal bars) of each variable (vertical axis) included in the Poisson regression model: symptomatic at presentation to care, living in a COVID-19 hotspot, hospital admission Neighborhood Deprivation Index and age

Three variables were predictors of enrollment: living in a COVID-19 hotspot [2.4 (1.8, 3.2)] (*p* < 0.0001), hospital admission [1.8 (1.2, 2.7)] (*p* = 0.002), and whether the participant was symptomatic at presentation to care [1.8 (1.2, 2.8)] (*p* = 0.006) (Fig. 1). Although age and Neighborhood Deprivation Index were hypothesized to affect enrollment, these factors were not significant predictors in the Poisson regression model.

### Predictors of biospecimen donation

Among people who enrolled, 79% donated any biospecimen; 97 individuals donated nasopharyngeal swabs, 119 donated oropharyngeal swabs, 105 donated blood, 16 donated urine, and 16 donated stool specimens (urine and stool were only collected from hospitalized patients) (Table 3). Among enrollees, living in a COVID-19 hotspot (*p* = 0.01), whether a COVID-19 test was conducted (*p* = 0.05), and positive COVID-19 test result (*p =* 0.02) were associated with donating nasopharyngeal specimens. Known epidemiological risk (*p =* 0.04) was associated with donating oropharyngeal specimens. Age, sex, race, ethnicity, and zip code-based Neighborhood Deprivation Index and other variables did not predict biospecimen donation.

**Table 3:** Characteristics of donated biospecimens (*N* = 204)

Specimen types	Significant variables	Count	(%)	P-value
Nasopharyngeal swab		97	(48)	
	Lives in COVID-19 hotspot			0.01
	Yes	41	(60)	
	No	56	(41)	
	COVID-19 test conducted			0.05
	Yes	81	(45)	
	No	16	(67)	
	COVID-19 test positive			0.02
	Yes	32	(58)	
	No	49	(39)	
Oropharyngeal swab		119	(58)	
	Epidemiological risk			0.04
	Yes	58	(52)	
	No	61	(66)	
Blood		105	(51)	
Urine		16	(8)	
Stool		16	(8)	

*IQR* interquartile range

We analyzed predictors among specimens with 20 or more samples. Urine and stool were only collected from hospitalized patients

## Discussion

In this study conducted at the beginning of the first surge of SARS-CoV-2 in greater Boston, Massachusetts, 47% of all individuals approached were willing to enroll in a pediatric biorepository for COVID-19. Individuals living in areas at high risk of COVID-19, with symptoms at presentation, or who were admitted to the hospital were the most likely to enroll. These factors may have contributed to a higher perceived risk in individuals that ultimately motivated them to enroll in the biorepository. Even though enrollees tended to be older than non-enrollees, all age categories of interest were represented among biorepository enrollees. Enrollment sites were among the few available places where children could be evaluated in person and tested for COVID-19 during the early pandemic. While we hypothesized that enrollment would be higher than in pediatric biorepositories in non-pandemic settings, these data are lacking for pediatric biorepositories with as broad a focus. Few studies have examined demographics and factors associated with declining enrollment at the time of approach for enrollment, either for pediatric clinical trial research [13], or pediatric biorepositories [14–17]. Furthermore, few existing studies on pediatric biorepositories enroll patients from a general pediatric population and rather focus on pediatric patients impacted by rare diseases such as congenital heart disease and cancer [13, 16, 18, 19]; enrollment rates in such biorepositories range from 55-97% [14, 18–21]. To our knowledge, this is the first study of demographic and epidemiologic predictors of enrollment in a pediatric biorepository examining characteristics of pediatric patients themselves (rather than their parents/guardians) or conducted in a pandemic setting [14, 15, 22].

Previous studies within and outside the US context have noted that parents willing to enroll their children in a biorepository are more likely to be highly educated, have a higher income, and experience more trust and positive attitudes towards clinical research [14, 17, 23]. Parents are generally willing to enroll children in clinical trials [24–26]. Parents agree to their children's participation of there is a higher perceived benefit to their children [15, 23]. Parents tend to overestimate the benefits of research on the well-being of their children and underestimate any risks in donating samples [26, 27]. While in the setting of this study, more deprived communities tended to be COVID-19 hotspots, it is notable that the community-level measurement of neighborhood socioeconomic status – Neighborhood Deprivation Index – did not predict enrollment. Participants and their parents may have had higher perceived risk in deprived neighborhoods, and therefore a tendency to agree to participate; furthermore, the establishment of community health research infrastructure antecedent to the pandemic may have facilitated research participation in these settings.

In the present study, parents were informed that donating samples would deepen our understanding of how COVID-19 impacts children. Although parents in the US and elsewhere may be less willing to enroll their children than themselves, parents’ beliefs related to their child’s enrollment generally mirror concerns they have for themselves [24, 27–32]. Most people are aware of the potential risks of biorepository participation but do not feel concern about being harmed [27]. Overall, pre-pandemic, people have reported high levels of trust in public health and clinical research institutions [16, 28, 30]. Systemic or institutional trust is critical to address common concerns about biorepository enrollment, including biospecimen misuse and unacceptable research in the future (specifically human cloning), risk of identification or identity theft, insurance discrimination, and potential for corporate exploitation [28–31]. Participants are comfortable donating if they feel they are adequately informed about how their biospecimens will be used. Those who feel the negative impact of biospecimens donation outweigh any positive impact tend to feel that people generally cannot be trusted, are most concerned about re-identification and have worries about information theft [30]. Concerns of data breaches and lack of confidentiality are deterrents to enrollment in biorepositories [33]. In certain studies, women and minorities are not as willing to donate their biospecimens, perhaps due to a historic lack of trustworthiness of institutions conducting clinical research (e.g., unconsented use of Henrietta Lacks’ cancer cells) [33–35]. In contrast to prior reports from countries like the US or Australia, in which parents who are white or non-immigrants are most comfortable enrolling children into biorepositories [14, 35], in this study, in which 44% identified as Hispanic, we found that no one racial or ethnic group was more likely to enroll than another.

We hypothesized that school-age children would be least likely to provide nasopharyngeal swabs and blood. Parents are less willing to enroll their children when they are concerned about the physical pain or discomfort that their children may face, if their children are more seriously ill, or if they are concerned about their children’s privacy and confidentiality [13, 15, 16]. We found no differences by age or other factors regarding participants’ willingness to donate biospecimens, which suggests that efforts in this population should be most focused on enrollment itself rather than consenting for specific biospecimen provision.

This study had important limitations. First, by design, this analysis was conducted by chart review and electronic medical record download. We were, therefore, unable to explore reasons for declining biorepository enrollment at the time of being approached; while there are many studies detailing parental willingness to participate in a pediatric biorepository, few have examined motivations for declining enrollment at the time of enrollment [14, 19]. Given reports of high willingness to enroll, there may be important gaps between intention to enroll, enrollment completion, and biospecimen donation. Qualitative surveys of parental perceptions of clinical research institutions, corporate exploitation, religiosity, and data breaches would bolster the interpretation of our study findings. Second, we lacked data on individual-level parental highest level of education and socioeconomic status; to address this, we incorporated Neighborhood Deprivation Index. We also did not collect information on religiosity; in one study lack of religiosity has been associated with willingness to donate biospecimens [32]; another study noted that some religious individuals consider their tissue to be 'sacred' or part of their identity and found that higher religiosity made parents less willing to consent their children for biorepository participation [16]. Finally, our analysis was conducted in Massachusetts and so we were not able to investigate how willingness to consent and enroll differed by state.

Living in a COVID-19 hotspot, the presence of symptoms at presentation, and hospital admission predicted a higher likelihood of pediatric biorepository enrollment among children with suspected or confirmed COVID-19 infection. While overall enrollment appeared to be low compared to previous reports of pediatric biorepository enrollment, the population who consented to biorepository participation reflected those seeking care: children of all age and Neighborhood Deprivation Index categories were represented, as well as patients recording Hispanic ethnicity, who comprised a major part of the first surge in Boston [36]. Understanding whether enrollment is representative of the COVID-19 pandemic is critical to interpreting analyses conducted on donated specimens. Furthermore, understanding factors related to non-enrollment may also permit research to encourage enrollment among key demographics.

## Supplementary Information

**Additional file 1.**

## Data Availability

The datasets used and/or analyzed during the study are not available publicly due to institutional requirements, however, are available from the corresponding author on reasonable request after following institutional requirements.

## Abbreviations

COVID-19: Coronavirus disease 2019
SARS-CoV- 2: Severe acute respiratory syndrome coronavirus 2
MGH: Massachusetts General Hospital
CDC: Centers for Disease Control and Prevention

## References

[1] Beers, Lee Salvio. American Academy of Pediatrics DC 2020 Letter. 202. Available from: https://downloads.aap.org/DOFA/AAP%20Letter%20to%20FDA%20on%20Timeline%20for%20Authorization%20of%20COVID-19%20Vaccine%20for%20Children_08_05_21.pdf. [cited 2021].

[2] Reopening Massachusetts | Mass.gov. 2020. Available from: https://www.mass.gov/info-details/reopening-massachusetts. [cited 2021].

[3] Centers for Disease Control and Prevention. COVID-19 weekly cases and deaths per 100,000 population by age, race/ethnicity, and sex. Available from: https://covid.cdc.gov/covid-data-tracker/#demographicsovertime. [cited 2021].

[4] Viner RM, Mytton OT, Bonell C, Melendez-Torres GJ, Ward J, Hudson L (2021). Susceptibility to SARS-CoV-2 infection among children and adolescents compared with adults: a systematic review and meta-analysis. JAMA Pediatr.

[5] Catchpoole DR, Carpentieri D, Vercauteren S, Wadhwa L, Schleif W, Zhou L (2020). Pediatric biobanking: kids are not just little adults. Biopreservation Biobanking.

[6] LaVergne SM, Stromberg S, Baxter BA, Webb TL, Dutt TS, Berry K (2021). A longitudinal SARS-CoV-2 biorepository for COVID-19 survivors with and without post-acute sequelae. BMC Infect Dis.

[7] The International Society for Biological and Environmental Repositories Presents Abstracts from Its 2021 Annual Meeting Connect and Collaborate through Biobanking: Powering Innovation and Discovery May 10–14, 2021. Biopreservation Biobanking 2021;19:A-1.

[8] Collins FS (2021). The long haul: forging a path through the lingering effects of COVID-19. Sect. House Energy amd Commerce Health Subcomittee.

[9] Lima R, Gootkind EF, De la Flor D, Yockey LJ, Bordt EA, D’Avino P (2020). Establishment of a pediatric COVID-19 biorepository: unique considerations and opportunities for studying the impact of the COVID-19 pandemic on children. BMC Med Res Methodol.

[10] Oster AM, Kang GJ, Cha AE, Beresovsky V, Rose CE, Rainisch G (2020). Trends in number and distribution of COVID-19 hotspot counties — United States, march 8–july 15, 2020. MMWR Morb Mortal Wkly Rep.

[11] Harvard Global Health Institute. Key metrics for COVID suppression: a framework for policy makers and the public. Edmond J. Safra center for Ethics; 2020. Available from: https://ethics.harvard.edu/files/center-for-ethics/files/key_metrics_and_indicators_v4.pdf. [cited 2021].

[12] Messer LC, Laraia BA, Kaufman JS, Eyster J, Holzman C, Culhane J (2006). The development of a standardized neighborhood deprivation index. J Urban Health Bull N Y Acad Med.

[13] Gao H, Jiang J, Feng B, Guo A, Hong H, Liu S (2018). Parental attitudes and willingness to donate children’s biospecimens for congenital heart disease research: a cross-sectional study in Shanghai. China BMJ Open.

[14] Antommaria AHM, Brothers KB, Myers JA, Feygin YB, Aufox SA, Brilliant MH (2018). Parents’ attitudes toward consent and data sharing in biobanks: a multisite experimental survey. AJOB Empir Bioeth.

[15] Paquette E, Shukla A, Davidson J, Rychlik K, Davis M (2019). Burden or opportunity? Parent experiences when approached for research in a pediatric intensive care unit. Ethics Hum Res.

[16] Qiu S, Song Y, Wang J, Gao P, Chen J, Chen R (2018). Factors that affect Chinese parents’ willingness to donate children’s biospecimens in pediatric research. Biopreservation Biobanking.

[17] Yamamoto M, Sakurai K, Mori C, Hata A. Participant mothers’ attitudes toward genetic analysis in a birth cohort study. J Hum Genet. 2021; Available from: http://www.nature.com/articles/s10038-020-00894-7. [cited 2021]. 10.1038/s10038-020-00894-7 PMC8225506 33495570

[18] Labib RM, Hassanain O, Alaa M, Ahmed S, Abou E-NS (2018). Planning today for tomorrow’s research: analysis of factors influencing participation in a pediatric cancer research biorepository. Front Oncol.

[19] Papaz T, Safi M, Manickaraj A-K, Ogaki C, Kyryliuk JB, Burrill L (2012). Factors influencing participation in a population-based biorepository for childhood heart disease. Pediatrics.

[20] Kong CC, Tarling TE, Strahlendorf C, Dittrick M, Vercauteren SM (2016). Opinions of adolescents and parents about pediatric biobanking. J Adolesc Health.

[21] Daljevic T, Safi M, Burill L, Dodge C, Chant-Gambacort C, Walter L-L, et al. Abstract 19926: pediatric participation in research: lessons learnt from a population-based cardiac biorepository. Circulation. 2010;122:A199266.

[22] Banks E, Herbert N, Mather T, Rogers K, Jorm L (2012). Characteristics of Australian cohort study participants who do and do not take up an additional invitation to join a long-term biobank: the 45 and up study. BMC Res Notes.

[23] Cunningham-Erves J, Deakings J, Mayo-Gamble T, Kelly-Taylor K, Miller ST (2019). Factors influencing parental trust in medical researchers for child and adolescent patients’ clinical trial participation. Psychol Health Med.

[24] Owen-Smith AA, Sesay MM, Lynch FL, Massolo M, Cerros H, Croen LA (2020). Factors influencing participation in biospecimen research among parents of youth with mental health conditions. Public Health Genomics.

[25] Murad AM, Myers MF, Thompson SD, Fisher R, Antommaria AHM (2017). A qualitative study of adolescents’ understanding of biobanks and their attitudes toward participation, re-contact, and data sharing. Am J Med Genet A.

[26] Klima J, Fitzgerald-Butt SM, Kelleher KJ, Chisolm DJ, Comstock RD, Ferketich AK (2014). Understanding of informed consent by parents of children enrolled in a genetic biobank. Genet Med.

[27] Lipworth W, Forsyth R, Kerridge I (2011). Tissue donation to biobanks: a review of sociological studies: tissue donation to biobanks: a review of sociological studies. Sociol Health Illn.

[28] Chan TW, Ho CW-L (2017). A ten-year retrospective analysis of consent for the donation of residual human tissue in a Singapore healthcare institution: reflections on governance. Asian Bioeth Rev.

[29] Simon CM, L’Heureux J, Murray JC, Winokur P, Weiner G, Newbury E (2011). Active choice but not too active: public perspectives on biobank consent models. Genet Med.

[30] Mello MM, Lieou V, Goodman SN (2018). Clinical trial participants’ views of the risks and benefits of data sharing. N Engl J Med.

[31] Lee SS-J, Cho MK, Kraft SA, Varsava N, Gillespie K, Ormond KE (2019). “I don’t want to be Henrietta lacks”: diverse patient perspectives on donating biospecimens for precision medicine research. Genet Med.

[32] Ahram M, Othman A, Shahrouri M, Mustafa E (2014). Factors influencing public participation in biobanking. Eur J Hum Genet.

[33] Goddard KAB, Smith KS, Chen C, McMullen C, Johnson C (2009). Biobank recruitment: motivations for nonparticipation. Biopreservation Biobanking.

[34] Skloot R (2010). The immortal life of Henrietta lacks.

[35] Savich RD, Tigges BB, Rios LI, McCloskey J, Tollestrup K, Annett RD (2020). Willingness of women to participate in obstetrical and pediatric research involving biobanks. J Community Genet.

[36] Bassett IV, Triant VA, Bunda BA, Selvaggi CA, Shinnick DJ, He W (2020). Massachusetts general hospital Covid-19 registry reveals two distinct populations of hospitalized patients by race and ethnicity. Camacho-Rivera M, editor. PLoS One.

