# Supplementary material for: Pediatric biorepository participation during the COVID-19 pandemic: predictors of enrollment and biospecimen donation
Source: BMC Pediatr. 2022 Mar 12;22:130. doi: 10.1186/s12887-022-03185-6 (PMC8917327; doi:10.1186/s12887-022-03185-6)
Supplement: Supplementary file 1 — Additional file 1. [file 12887_2022_3185_MOESM1_ESM.docx]

**SUPPLEMENTAL APPENDIX**

Pediatric biorepository participation during the COVID-19 pandemic: predictors of enrollment and biospecimen donation

Neilan *et al.*

**TABLE OF CONTENTS**

Supplemental Methods

eTable 1. Summary of model comparison results

**SUPPLEMENTAL METHODS**

**COVID-19 hotspot**

The United States (US) Centers for Disease Control and Prevention (CDC) defines a COVID-19 hotspot county as meeting all of the following four criteria: 1) >100 incident COVID-19 cases in the most recent week, 2) an increase in the most recent week’s COVID-19 incidence over the preceding week’s incidence, 3) a decrease of less than 60% or an increase in the most recent 3-day COVID-19 incidence over the preceding 3-day incidence, and 4) the ratio of 7-day incidence over 30-day incidence is greater than 0.31. In addition, hotspots must also meet at least one of the following criteria: 1) >60% change in the most recent 3-day COVID-19 incidence, or 2) >60% change in the most recent 7-day incidence.^1^

**COVID-19 risk level**

The COVID-19 risk level variable was created according to the key metrics for COVID-19 suppression framework developed by the Harvard Global Health Institute and Harvard’s Edmond J. Safra Center for Ethics.^2^ Risk levels were calculated based on the daily new cases per 100,000 people: green (<1), yellow (1-9), orange (10-24), red (≥25). The green level corresponds to the CDC’s low incidence threshold. We classified the color coding as low risk (green), mild risk (yellow), moderate risk (orange), and high risk (red).

**Neighborhood Deprivation Index**

We used the method described by Messer *et al.* to calculate the Neighborhood Deprivation Index (NDI).^3^ We used data from the American Community Survey from the U.S. Census to create the NDI.^4^ Accounting for the specific context in which the study was conducted, we identified twenty-five variables that we deemed to be related to the neighborhood-level socioeconomic status and deprivation level. Of the included variables, twenty variables were initially used by Messer *et al.* We identified the remaining five variables from the literature (percent of nonessential workers, population density per square mile, percent insured, median household income in thousands, and average household size). As described elsewhere, we used principal component analysis (PCA) to calculate the standardized first principal component. Variables retained fulfilled two criteria: 1) if the variable had a loading above 0.25, and 2) if the lower 95% confidence limit of the variable loading was not below the lower 95% confidence limit for the median variable loading. Sixteen variables were retained based on the first criteria, and eight fulfilled the second criteria (lower limit of the 95% CI =0.54). Therefore, in the current analysis, we retained eight variables in the final NDI (percent males and females with more than a bachelor’s degree level education, percent males in management, percent males in professional occupations, percent females in management, percent females in professional occupations, percent of nonessential workers, percent insured, and the median household value in thousands). The estimated NDI is a standardized score, that has a mean of zero and standard deviation of one. Higher values of NDI indicated a more affluent “less deprived” neighborhood (i.e., a neighborhood that has an NDI score of 0.8 is less deprived than a neighborhood that has an NDI score 0.2). We also classified neighborhoods based on the median value of NDI. Neighborhoods with NDI scores less than the median was classified as “more deprived”, and neighborhoods with NDI scores more than the median was classified as “less deprived.”

**Poisson regression model selection**

To select final multivariable Poisson models, we combined information gained from two approaches: 1) fitting and selecting from sets of a priori models, and 2) automatic stepwise selection. We first started with an a priori list of variables to be tested in the bivariate association. The a priori list was based on epidemiologic and clinical characteristics that we deemed as important characteristics that were plausibly related to enrollment. Subsequently, we started the model selection by running a saturated model by including all possible exposure variables (the a priori list) that did not cause convergence problems due to collinearity or being in the causal pathway of another proximal predictor and the outcome. Subsequently as a priori, we planned and fitted several reduced models including: a model that includes only significant predictors from the bivariate association, a model that included only significant demographic predictors, a model that included only significant clinical predictors, a model that included predictors selected by the automatic selection, and a model that included any variable that was significant in any of the reduced models. Our final selected model included demographic and clinical predictors that, based on epidemiologic and clinical knowledge, were the most appropriately and meaningfully selected during the model selection process. To assess goodness of fit, non-nested and nested models were compared using Akaike information criterion (AIC). The lower the AIC the better the model fit (eTable 1).

**REFERENCES**

1. Oster AM, Kang GJ, Cha AE, Beresovsky V, Rose CE, Rainisch G *et al.* Trends in number and distribution of COVID-19 hotspot counties — united states, march 8–july 15, 2020. *MMWR Morb Mortal Wkly Rep* 2020; **69**: 1127–1132.
2. Harvard Global Health Institute. Key metrics for COVID suppression: a framework for policy makers and the public. 2020. https://ethics.harvard.edu/files/center-for-ethics/files/key_metrics_and_indicators_v4.pdf (accessed 30 Apr 2021).
3. Messer LC, Laraia BA, Kaufman JS, Eyster J, Holzman C, Culhane J *et al.* The development of a standardized neighborhood deprivation index. *J Urban Health Bull N Y Acad Med* 2006; **83**: 1041–1062.
4. Bureau UC. American community survey 5-year data (2009-2019). The United States Census Bureau. https://www.census.gov/data/developers/data-sets/acs-5year.html (accessed 9 Jul 2021).

**eTable 1. Summary of model comparison results**

| Models compared | AIC |
| --- | --- |
| Full model | 752.98 |
| Automatic selection | 686.43 |
| Planned selection |  |
| Reduced model 1 | 716.31 |
| Reduced model 2 | 716.45 |
| Reduced model 3 | 754.93 |
| Reduced model 4 | 706.62 |
| Reduced model 5 | 712.87 |
| Reduced model 6 | 709.55 |
| Reduced model 7 (Final model) | 709.64 |

AIC, Aikake information criterion

Full model: Poisson regression with predictors that did not cause convergence issues

Reduced model 1 included only significant predictors from bivariate association.

Reduced model 2 included only significant demographic predictors from bivariate association.

Reduced model 3 included only significant clinical predictors from bivariate association.

Reduced model 4 included significant variables from Poisson regression with automatic selection.

Reduced model 5 involved significant variables from the full model.

Reduced model 6 included any significant variables identified from the previous models.

Reduced model 7, our final model, involved demographic and clinical predictors that were most appropriate and meaningful during the model selection process.
